# Isolated Rearing at Lactation Increases Gut Microbial Diversity and Post-weaning Performance in Pigs

**DOI:** 10.3389/fmicb.2018.02889

**Published:** 2018-11-29

**Authors:** Tsungcheng Tsai, Marites A. Sales, Haejin Kim, Gisela F. Erf, Nguyen Vo, Franck Carbonero, Marie van der Merwe, Elizabeth B. Kegley, Randy Buddington, Xiaofan Wang, Charles V. Maxwell, Jiangchao Zhao

**Affiliations:** ^1^Department of Animal Science, Division of Agriculture, University of Arkansas, Fayetteville, AR, United States; ^2^Department of Poultry Science, Division of Agriculture, University of Arkansas, Fayetteville, AR, United States; ^3^Department of Food Science, Division of Agriculture, University of Arkansas, Fayetteville, AR, United States; ^4^School of Health Studies, University of Memphis, Memphis, TN, United States

**Keywords:** isolated rearing, neonatal pigs, swine gut microbiome, growth performance, adaptive immune cells

## Abstract

Environment and diet are two major factors affecting the human gut microbiome. In this study, we used a pig model to determine the impact of these two factors during lactation on the gut microbiome, immune system, and growth performance. We assigned 80 4-day-old pigs from 20 sows to two rearing strategies at lactation: conventional rearing on sow’s milk (SR) or isolated rearing on milk replacer supplemented with solid feed starting on day 10 (IR). At weaning (day 21), SR and IR piglets were co-mingled (10 pens of 4 piglets/pen) and fed the same corn-soybean meal-dried distiller grain with solubles- and antibiotic-free diets for eight feeding phase regimes. Fecal samples were collected on day 21, 62, and 78 for next-generation sequencing of the V4 hypervariable region of the bacterial 16S rRNA gene. Results indicate that IR significantly increased swine microbial diversity and changed the microbiome structure at day 21. Such changes diminished after the two piglet groups were co-mingled and fed the same diet. Post-weaning growth performance also improved in IR piglets. Toward the end of the nursery period (NP), IR piglets had greater average daily gain (0.49 vs. 0.41 kg/d; *P* < 0.01) and average daily feed intake (0.61 vs. 0.59 kg/d; *P* < 0.01) but lower feed efficiency (0.64 vs. 0.68; *P* = 0.05). Consequently, IR piglets were heavier by 2.9 kg (*P* < 0.01) at the end of NP, and by 4.1 kg (*P* = 0.08) at market age compared to SR piglets. Interestingly, pigs from the two groups had similar lean tissue percentage. Random forest analysis showed that members of *Leuconostoc* and *Lactococcus* best differentiated the IR and SR piglets at weaning (day 21), were negatively correlated with levels of Foxp3 regulatory T cell populations on day 20, and positively correlated with post-weaning growth performance. Our results suggest that rearing strategies may be managed so as to accelerate early-life establishment of the swine gut microbiome to enhance growth performance in piglets.

## Introduction

Modern genetic selection has dramatically increased swine litter size. However, increased litter size also leads to higher pre-weaning piglet mortality (17.3% in 2016 vs. 14.2% in 2008) (Knauer and Hostetler, 2013; Stalder, 2017), which is mainly attributed to sow crushing and starvation from insufficient nutrients. Malnutrition of piglets often results in immunodeficiency (Bourke and Berkley, 2016), abnormal body thermoregulation (Muns et al., 2016), increased piglet birth weight variation (Milligan et al., 2002; Fix et al., 2010; Ferrari et al., 2014), and lightweight weanlings (Fix et al., 2010; Baxter et al., 2013), which compromise the well-being of neonatal pigs and sows and ultimately result in production losses.

Isolated rearing (IR), a strategy wherein neonatal piglets are separated from the sows, has been applied to reduce mortalities caused by the sow crushing of piglets. However, like other mammalian species, pigs rely on colostrum to receive passive immunity from the dam (Wijtten et al., 2011) with expression of maternal antibody transferring Fc receptors in the gastrointestinal tract (GIT) during the first several hours postpartum. In addition to providing nutrients, colostrum and milk are also critical for the development of digestive enzymes (Edwards and Parrett, 2007) and play an important role in stimulating and training immune system development (Mazmanian et al., 2005). Recent data demonstrate that the commensal microbiota is integral in the development of the immune system (Kau et al., 2011; Shibata et al., 2017). Depriving piglets of the beneficial effects of sow’s milk has been linked to poor growth performance during the nursery stage in IR piglets after co-mingling with their sow reared (SR) counterparts (Main et al., 2004; Davis et al., 2006; Cabrera et al., 2010; Turfkruyer and Verhasselt, 2015).

The GIT is home to trillions of microorganisms, most of which are bacteria that have co-evolved with the host and play important roles in nutrient processing and absorption (Kau et al., 2011). Many factors, including genetics, diet, environment, age, and antibiotics, have been reported to affect the human gut microbiome (Cho and Blaser, 2012). How these factors affect the swine gut microbiome has become a subject of investigation in recent years (Mulder et al., 2009; Schmidt et al., 2011; Looft et al., 2012; Schokker et al., 2014; Chen et al., 2017, 2018; Xiao et al., 2018). In the present study, we were particularly interested in the effect of rearing environment and diet on the swine gut microbiome and consequently, on growth performance. Thus, we reared piglets 4 day postpartum by isolation in a new environment (isolation decks). We provided these piglets with bovine milk replacer and later, starting on day 10, with solid feed. We observed significantly increased microbial diversity in the IR piglets compared to their SR siblings. Moreover, IR piglets survived co-mingling process and performed better than SR piglets after weaning. Our study suggests that IR coupled with solid feed is an effective rearing strategy to modulate the swine gut microbiome and improve post-weaning growth performance in swine.

## Results

### Isolated Rearing Increased Post-weaning Growth Performance

**FIGURE 1:**
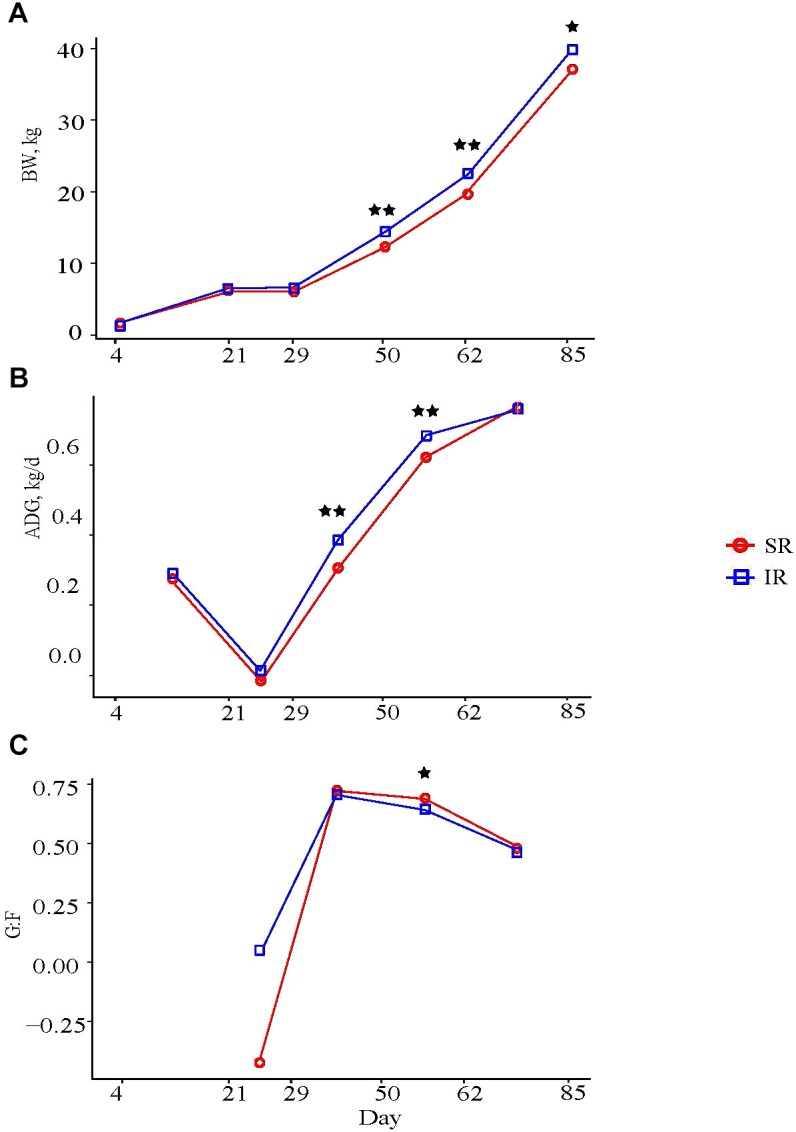
Effect of rearing environment and source of nutrients during lactation on **(A)** body weight (BW, kg), **(B)** average daily gain (ADG, kg/d), and **(C)** feed efficiency of pigs (4 to 85-day-old, least square means). Four littermates were assigned to two treatments [SR (red), sows reared; IR (blue), isolated reared] 4 days postpartum from 20 sows with two neonates transferred to an offsite nursery facility and housed in deck while 2 littermates remained with sows. Upon weaning at day 21, all pigs were transferred into the same facility and fed the same diets from weaning to study completion, with IR pigs housed in adjacent pens to SR littermates. Data were analyzed using GLM procedure of SAS with the two stars symbol indicating significant treatment differences (*P* < 0.01) and the single star symbol showing a tendency of significance (0.05 < *P* ≤ 0.10).

Isolated rearing pigs were heavier than SR pigs from day 50 through the end of the remaining trial, even though mean BW at weaning was similar between both groups, (Figure 1A); at study completion, IR pigs tended to be heavier than SR pigs (Supplementary Table S1, 136.37 vs. 132.24 kg, *P* = 0.08). During the nursery period (NP), IR pigs grew faster than SR pigs at NP2 (average daily gain, ADG, day 29–50: 0.38 vs. 0.30 ± 0.015 kg/d; *P* < 0.01) and NP3 (day 50–62: 0.68 and 0.61 kg/d; *P* < 0.01; Figure 1B). These results are consistent with a greater average daily feed intake (ADFI) in IR compared to SR pigs at NP1 (0.16 vs. 0.10 kg/d; *P* < 0.01) and NP2 (0.51 vs. 0.41 kg/d; *P* < 0.01; Supplementary Table S2), whereas a tendency for higher intake was observed in IR pigs than those in SR pigs (1.01 vs. 0.91 kg, *P* = 0.10) at NP3. Feed efficiency (weight gain over intake, G:F) in IR pigs tended to be lower than that in SR pigs at NP3 (0.64 vs. 0.68; *P* = 0.05; Figure 1C), but was not different between the two groups overall (0.36 vs. 0.37; *P* > 0.73; Supplementary Table S2).

As for carcass composition, IR pigs had deeper 10th-rib back fat (23 vs. 20 mm; *P* < 0.05) and larger 10th rib longissimus muscle area (49.5 vs. 47.2 cm^2^; *P* = 0.08) than SR pigs, but both groups had similar lean tissue percentages (38.2 vs. 38.5%; Supplementary Table S2).

### Isolated Rearing Increased Microbial Diversity and Altered Microbiome Structure

**FIGURE 2:**
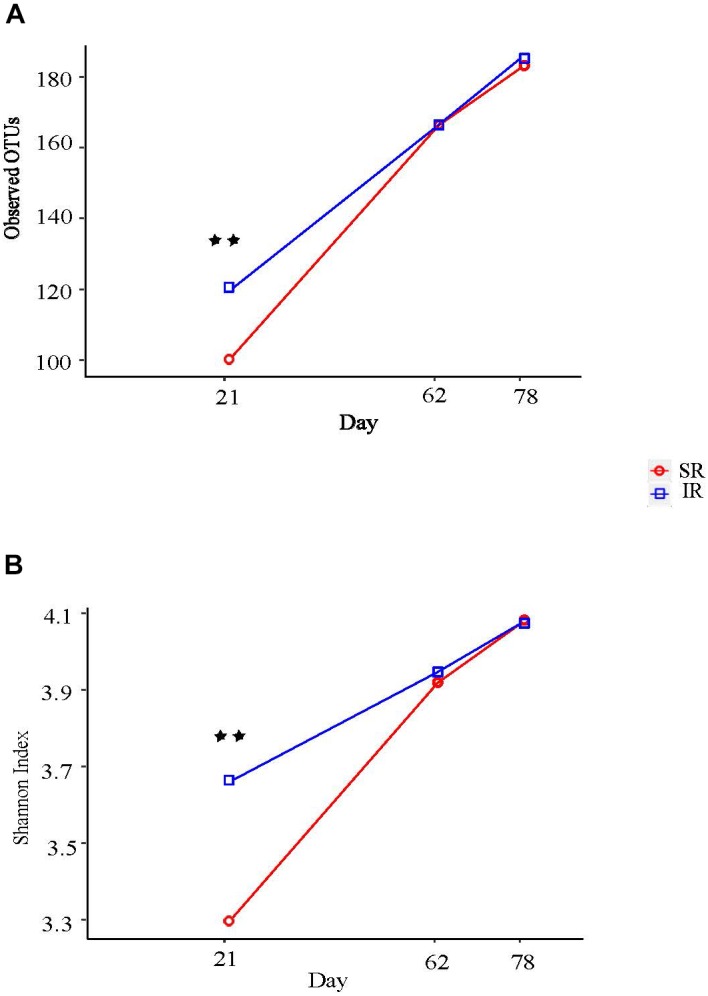
Effect of different rearing environment and source of nutrients during lactation on alpha diversity of fecal samples collected from 21, 62, and 78 day old pigs. Microbial alpha diversity was calculated with observed operational taxonomic units (OTUs) **(A)** and Shannon index **(B)**. Significant differences between groups were determined by nonparametric Wilcoxon Rank Sum test. SR, sows reared; IR, isolated reared. ^∗∗^Indicates significant difference between treatments (*P* < 0.01).

**FIGURE 3:**
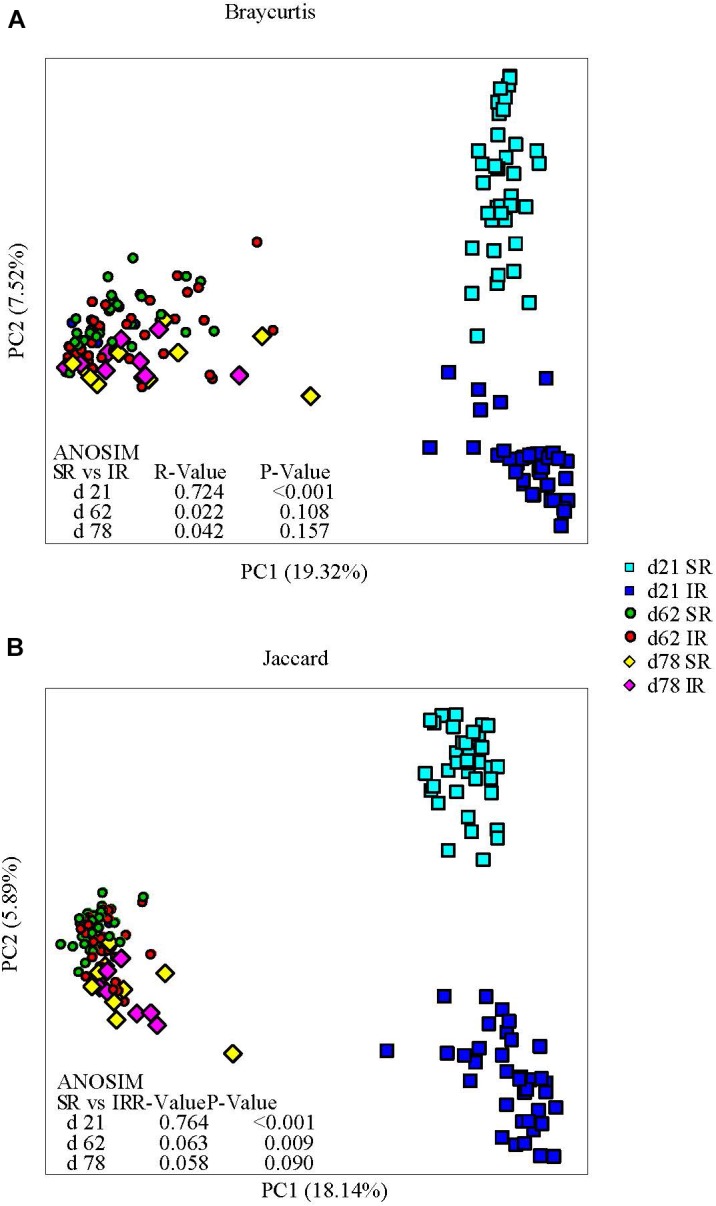
Effect of rearing environment and source of nutrients during lactation on gut microbiome structure (**A**, Bray-curtis) and membership (Jaccard, **B**) demonstrated by principal coordinates (PC) analysis plots. Stool samples collected from days 21, 62, and 78 were illustrated by squares, circles and diamonds, respectively. The analysis of similarity (ANOSIM) procedure was used to test the statistical significance between groups. SR, sows reared; IR, isolated reared.

Isolated rearing increased both richness (observed number of operational taxonomy units [OTUs]) and microbial diversity (indicated by a greater Shannon diversity index) on day 21 (end of lactation). Differences in alpha diversity measures diminished post weaning, as shown on day 62 and 78, after both pig groups were co-mingled and fed the same diets (Figures 2A, B). With respect to beta diversity, principal coordinates analysis (PCoA) based on Bray-Curtis dissimilarities (ANOSIM, *R* = 0.724, *P* < 0.01; Figure 3A) and Jaccard distance (ANOSIM, *R* = 0.764, *P* < 0.01; Figure 3B) demonstrated that IR and SR pigs had significantly different gut microbiome membership and structure on day 21. Consistent with our findings on alpha diversity, differences in community membership and structure also disappeared on day 62 (ANOSIM, *R* = 0.022, *P* = 0.108) and day 78 (ANOSIM, *R* = 0.157, *P* = 0.142) after co-mingling.

**FIGURE 4:**
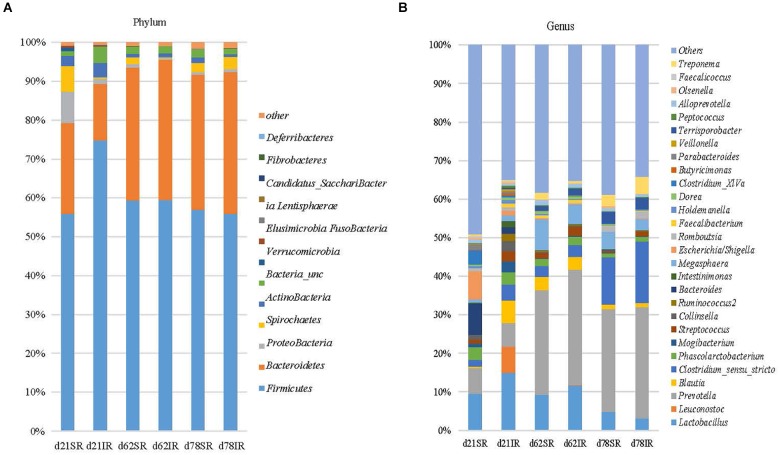
Stacked bar chart showing the mean relative abundance of top 12 phyla **(A)** and top 39 genera **(B)** across age (day 21, day 62, and day 78) between treatments (SR, sow reared; IR, Isolated reared) covering 99% (phyla) and 51–66% (genera) of the sequence, respectively.

Regarding gut microbiome composition on day 21, *Bacteroidetes* (23.4% vs. 14.5%), *Proteobacteria* (7.9% vs. 1.4%), and *Spirochaetes* (6.7% vs. 0.4%) were more abundant in SR pigs than in IR pigs at the phylum level (Figure 4A) whereas *Firmicutes* (56.0% vs. 74.8%), and *Actinobacteria* (2.5% vs. 3.7%) were lower in SR pigs than their IR littermates at weaning (day 21). At the genus level, *Leuconostoc* (6.8% vs. 0.003%)*, Blautia* (5.86% vs. 0.29%), *Intestinimonas* (1.6% vs. 0.3%)*, Clostridium_sensu_stricto* (4.13% vs. 1.72%)*, Mogibacterium* (2.78% vs. 0.95%) and *Ruminococcus2* (*Lachnospiraceae*; 1.91% vs. 0.04%) were more abundant in IR pigs while *Bacteroides* (1.72% vs. 8.06%)*, Escherichia/shigella* (1.07% vs. 7.30%), and *Clostridium XIVa* (0.58% vs. 3.72%) were overrepresented in SR pigs on day 21 (Figure 4B). On day 62, SR pigs had greater *Treponema* (1.75% vs. 0.55%) than IR pigs. In addition, *Lactobacillus* was more abundant in SR pigs in both day 21 and day 62 than IR pigs.

### Isolated Rearing Enriched Potentially Beneficial Bacteria

**FIGURE 5:**
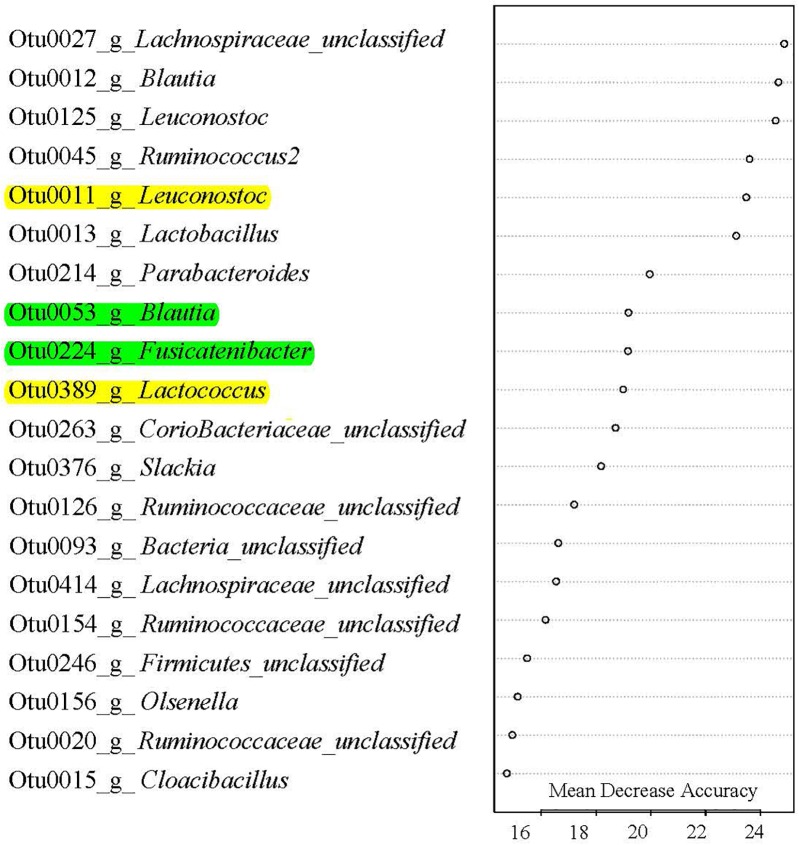
Gut microbes signature of rearing environment and nutrient resources. Top 20 most predictive OTUs that differentiate SR (sow reared) from IR (isolated reared) in 20-day-old pigs. These OTUs were ranked by random forest based on their Mean Decrease Accuracy. Out-of-bag error rate was 0%. A single letter following OTU number indicates genus, which was classified against the Ribosomal Database Project. All OTUs except OTU15 Cloacibacillus were more abundant in IR pigs than SR pigs. Of the top 20 OTUs, those highlighted with yellow color were negatively correlated with day 20 Foxp3 Treg T cell population, and positively correlated with subsequent growth phenotypes, while those OTUs highlighted with green color were positive correlated with growth performance (see Supplementary Table S3 for details).

Classification-based random forest analysis identified the top 20 OTUs that are the most predictive of the treatment (Figure 5) on day 21. Specifically, OTUs associated with *Fusicatenibacter*, *Blautia*, *Leuconostoc*, *Lactococcus*, and *Lactobacillus* were enriched in IR piglets. Interestingly, regression-based random forest analysis showed that these OTUs positively correlated with subsequent growth performance. Positive correlations were observed between: OTU53 (*Blautia*) and ADG on day 21 to 29 (Supplementary Table S3); OTU11 (*Leuconostoc*) and ADG on day 29 to 50 and day 21 to 62 (Supplementary Table S3); OTU224 (*Fusicatenibacter*) and BW on day 50 and 62 (Supplementary Table S3); and OTU389 (*Lactococcus*) and BW on day 50 and ADG on day 29 to 50 (Supplementary Table S3). On the other hand, some of the OTUs negatively correlated to Foxp3^+^ T cell concentration in blood on day 21 including OTU11 (*Leuconostoc;* Supplementary Table S3), and OTU389 (*Lactococcus;* Supplementary Table S3).

### Isolated Rearing Decreased Lymphocyte Expansion

**FIGURE 6:**
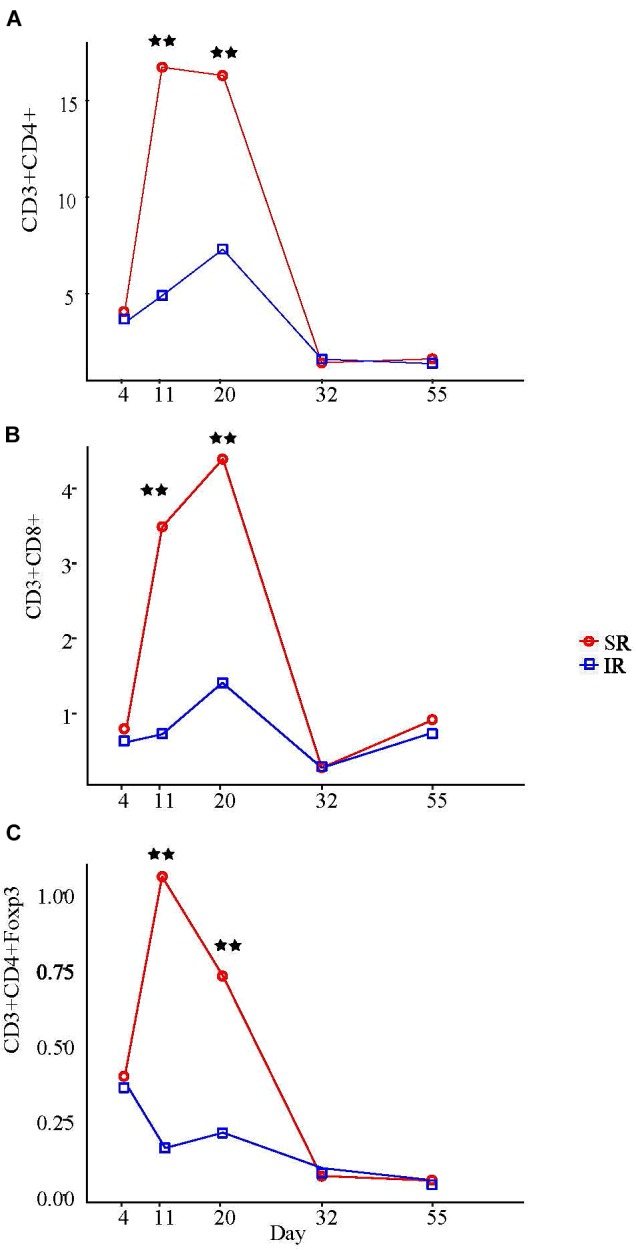
Effect of rearing environment and source of nutrients during lactation on absolute CD3+CD4+ T helper cells **(A)**, CD3+CD8+ cytotoxic T cells **(B)**, and CD3+CD4+Foxp3+ regulatory T cells **(C)** in blood [×10^6^/ml] in 4-, 11-, 20-, 32-, and 55-day-old pigs (LS means). One pig from each litter (10 litter per treatments) was selected for blood sampling. The same pigs were sampled throughout the entire study. The whole blood samples (3 mL) were obtained at day 4 (initial), 11, 20 (weanling), 32, 55, and 103 of ages. PBMCs were isolated using Histopaque^®^1077 (Sigma-Aldrich, St. Louis, MO, United States) density gradient centrifugation, counted by hemocytometer, and expressed as blood concentration based on the volume of whole blood used to isolate the PBMCs, the total number of PBMCs isolated, and the proportions of lymphocyte subsets determined by immunofluorescent staining and flow cytometry. Results on day 103 are presented in Supplementary Table S4. Two stars refer to ages having significant differences between treatments when compared to other ages at probability value <0.01.

Among all the peripheral blood leukocyte populations quantified by flow cytometry, only T lymphocyte populations showed significant treatment × age interaction (*P* < 0.01; Supplementary Table S4). On day 11 and 20, SR piglets had more helper T cells (T_H;_ CD3^+^CD4^+^CD8^−^) and cytotoxic T cells (T_C_; CD3^+^CD4^−^CD8^+^) than IR littermates (Figures 6A, B). Specifically, although T_H_ cell counts in both pig groups began increasing after day 4, SR pigs had almost four times more T_H_ cells than IR pigs a week later (16.6 vs. 4.8 [×10^6^/ml]; Figure 6A). While sustained in SR pigs on day 20, T_H_ cell number peaked in IR pigs, although numbering just half as many as those in SR pigs (16.2 vs. 7.2 [×10^6^/ml]; *P* < 0.01). On the other hand, while the T_C_ cell number in SR pigs started increasing after day 4, that of IR pigs began rising a week later and peaked on day 20, albeit only half the number in SR piglets (1.5 vs. 4.7 [×10^6^/ml]; Figure 6B). The regulatory T (T_reg_) cell fraction of the T_H_ (CD4+) cell population followed a similar pattern (Figure 6C). Lowest points for all T cell populations in both pig groups were at day 32; any increase in cell concentration thereafter was minimal and did not surpass peak points on day 11 day 20.

## Discussion

The primary objective of this research was to examine whether rearing environment and nutrient resource at lactation impact swine post-weaning performance, gut microbiome and immune system development.

Although isolated rearing has been practiced to decrease sow crushing mortalities, contradictory results have been reported regarding its effect on piglet mortality. Two independent studies (Patience et al., 2000) showed that pigs weaned to a clean isolated facility at 12 days of age exhibited improved growth rate, likely attributed to reduced exposure to infectious agents (Fangman et al., 1996). However, pigs in these reports were not co-mingled with pigs raised on a conventional farm. In contrast, some studies demonstrated that weaning at an early age decreased performance (Cabrera et al., 2010), increased mortality rate (Main et al., 2004), lowered total white blood cell and lymphocyte concentrations (Davis et al., 2006), and exacerbated immune activity upon pathogen challenge (McLamb et al., 2013). Our results suggest that co-mingling with SR pigs post-weaning did not alter immune cell populations nor negatively affect post-weaning performance in IR pigs. Possible explanations for these contradicting findings in our study are: (1) pigs in our swine facility did not experience pathogenic challenges of a magnitude comparable to those in a large commercial swine production setting, thus nutrients were likely partitioned more toward gastrointestinal tract development (Tang et al., 2013) rather than to immune responses; (2) Supplementation of solid feeding in IR pigs at 10–20 days of age led to accelerated development of the gut microbiota, which might contribute to better adaptation to post-weaning changes in the diet and environment

Supplement milk replacer and solid feed (solid feed supplement while animals are still nursing) have been associated with expedited intestinal adaptation to solid diets (Cabrera et al., 2013), counteracting the deleterious impact of early weaning from dams on the morphological and functional properties of the small intestine (Pluske et al., 1997), and improving growth performance of piglets (Pustal et al., 2015). In addition, early exposure to soy protein in neonates has been shown to stimulate antibody production (Bailey et al., 1993, 1994). This response was limited at the mucosal rather than the systemic level and was attenuated in subsequent antigen exposure. This suggests that oral tolerance to food antigens in IR pigs (with small expansion of T cells on day 20) could be the reason for their superior post-weaning performance. Early introduction of solid feed also enabled the IR piglets to adapt to dietary changes post-weaning faster than their SR littermates. In humans, evidence suggests that early solid food introduction and increased variety of food choice in the 1st year of life attenuates sensitivity to food and respiratory allergens later in life, and lowers the risk of atopic diseases (Zutavern et al., 2004; Nwaru et al., 2014; Roduit et al., 2014). It has been reported in humans that introduction of solid food significantly increased gut microbiome diversity, short chain fatty acids (SCFAs) production, and genes associated with carbohydrate utilization and vitamin biosynthesis in infants (Koenig et al., 2011). Little is known about the effect of milk replacer and creep feed on the succession of the swine gut microbiome. Gaorui et al. (2016) showed that solid feed and weaning are likely the major factors affecting the establishment of the composition and diversity of the gut microbiota in pigs after weaning. Our study suggests that isolated rearing and the addition of solid feed to the neonatal pigs’ diet accelerated the maturation of the swine gut microbiome through increased microbial diversity and enrichment of several bacterial taxa, members of which are known SCFA producers. This expedited gut microbiome establishment not only hastened the piglets’ adaptation to the post-weaning diet during the nursery phase but also reduced the Foxp3 regulatory T cell number cells in the IR pigs during lactation. A relatively larger magnitude of peripheral T_reg_ expansion in sow-reared pigs was observed in our study, consistent with the study by Lewis et al. (2012). Thymic derived CD4^+^Foxp3^+^ T_reg_ cells are mainly responsible for the maintenance of tolerance to self antigen (Povoleri et al., 2013), while peripheral induced T_reg_ cells are important for minimizing inflammatory responses against dietary antigen and commensal microbiota to prevent excessive tissue injury and to enable tissue repair (Izcue et al., 2006).

The expansion of T cells in SR pigs was negatively correlated with several bacterial taxa at weaning: *Leuconostoc, Blautia, Ruminococcaceae, and Fusicatenibacter*. Members of *Blautia* and *Ruminococcaceae* are SCFAs producers (Louis and Flint, 2017), and SCFAs serve as primary energy source for colon epithelial cells. More interestingly, SCFAs have anti-inflammatory properties and regulate immune responses. It is likely that these SCFAs may have played some role(s) in reducing the immune responses (i.e., by decreasing inflammation) in the IR piglets during lactation. The immune response can increase the metabolic rate which demands energy and nutrients in the event of environmental stress, such as a high pathogen load in the gut, diminishing resources that would otherwise be spent on physiological processes such as growth or muscle accretion in the absence of an immune challenge (Ganeshan and Chawla, 2014). Even a mild immune reaction is enough to suppress feed intake and growth (Lochmiller and Deerenberg, 2000). Our results generally agree with published data on suboptimal growth performance in animals with highly stimulated immune systems (Williams et al., 1997). We speculate that, given a relatively better post-weaning performance in IR pigs, low immune stimulation in IR pigs during lactation allowed them to repartition nutritional resources toward growth rather than for immune responses.

Of note, there are some limitations of study. First, the increased post-weaning growth performance of the IR pigs might be a result of the combined effects of a cleaner environment and different diet (bovine milk plus solid feed). More experiments are needed to tease apart their separate effects. For example, two more treatment groups (bovine milk replacer without solid feed vs. swine milk replacer plus solid feed) would enable us to determine the effects of different types of milk replacer and the solid feed on the swine gut microbiota and post-weaning growth performance. Second, we did not sample the gut microbiota before the treatment and thus we don’t know the variation of the baseline swine gut microbiota. Given the fact that we selected the same number of littermates, with two piglets separated from and two piglets staying with the sows, we assume the baseline variation between the treatment and the control group will be minimal, if any. In addition, more sampling points are also needed to examine the rate of divergence and convergence of the swine gut microbiome affected by isolated rearing and by the co-mingling of the two groups after treatment. Finally, we did not assess animal welfare in the early-weaned piglets. It is expected that early weaning from the sows would result in remarkable post-weaning stress to the treatment group. However, we did not observe any difference in mortality or morbidity between the IR and the SR piglets, suggesting that the early weaning stress might have been compensated for the combined effect of a cleaner environment and a different diet.

In summary, our data indicate that isolated rearing with solid feed supplementation significantly increased swine gut microbial diversity, enriched beneficial bacteria and increased growth performance. Further experiments such as fecal matter transplant from IR pigs to SR pigs are warranted to determine whether the enhanced animal productivity was a direct consequence of these changes in the swine gut microbiome in an experimental setting.

## Materials and Methods

All experimental procedures involving animals were approved by the University of Arkansas Institutional Animal Care and Use Committee (IACUC# 13060).

### Animals and Treatments

Animal experiments were conducted at the University of Arkansas-Division of Agriculture Swine Research Unit in Fayetteville, Arkansas. Approximately 4 days postpartum, a total of 80 piglets (PIC-C 29 × 380 pigs) were selected from 20 litters with four pigs/litter, blocked by body weight (BW), and randomly assigned to one of two rearing strategies during lactation: (1) isolated rearing (IR) on deck or (2) sow rearing (SR) where two littermates remained with the sow and other siblings in the farrowing crate. No significant difference in the initial BW was observed between the SR (*n* = 40, 1.24 ± 0.04 kg) and the IR pigs (*n* = 40, 1.27 ± 0.21 kg). During lactation, SR piglets received only sow’s milk, whereas IR pigs were fed with bovine milk replacer (Birthright^TM^) supplemented with antibiotic-free solid feed (nursery phase 1 diet) starting at day 10 according to manufacturer’s instructions (Ralco Nutrition, Inc.). Isolation decks were installed in an offsite nursery facility, which was approximately 10 miles away from the sow barns. Each isolation deck (1.41 m × 0.86 m) contained two milk cups and two heat lamps. Animals were managed according to manufacturer’s instructions (Birthright Deck^TM^, Ralco Nutrition Inc.). Ambient temperature was set at 30°C.

Upon weaning (day 21), both SR and IR pigs were blocked by gender, and lactation treatments and transferred to the same conventional nursery facility where two litters from the same rearing strategy were co-mingled (i.e., two pigs/two litters from each strategy) in one pen, and pigs from different lactation treatments were housed in adjacent nursery pens to allow maximum exposure between two groups. At the end of nursery, all pigs were moved to wean-to-finish facility and pigs remained with their penmates from weaning until study completion. All pigs were fed corn-SBM-DDGS-antibiotic-free diets formulated to meet or exceed nutrient requirements (PIC, 2011) for the three-phase nursery period (NP 1: day 21–29; NP 2: day 29–50; NP 3: day 50–62) and five-phase growing-finishing (grower phase [GP] 1: day 62–85; GP 2: 85–119; finisher phase [FP] 1: day 119–141; FP 2: day 141–159; FP 3: day 159–181) feeding regimens (Supplementary Table S5).

### Gut Microbiota Analyses

Fecal samples were collected on days 21, 62, and 78 and stored at −80°C until processed for next-generation sequencing targeting the V4 hypervariable region of the 16S rRNA gene following the procedure in Vo et al. (2017). Briefly, genomic DNA was extracted from fecal samples using the DNeasy stool kit (Qiagen, Inc., West Carlsbad, CA, United States), quantified with NanoDrop (ThermoFisher Scientific, Waltham, MA, United States), normalized to 10 ng/μl, and used as template for 16S rRNA gene sequencing on the Illumina Miseq platform using the MiSeq Reagent kit v2 (Illumina, Inc., San Diego, CA, United States). Sequences were analyzed by using the mothur (1.34.0) software package to eliminate sequence error and chimeras following the procedure from Miseq SOP^1^. Sequences were aligned to SILVA reference alignment (Release 128) specific for the V4 region, assigned to different operational taxonomic units (OTUs) with 97% similarity, and classified using the RDP Bayesian classifier. Sequences from each sample were normalized to 1,000 to minimize the effect of sequencing depth on alpha and beta diversity analysis. The effect of environment and diet on the swine gut microbiome were evaluated by (1) alpha diversity (e.g., Shannon diversity index and observed number of OTUs); and (2) Bray-Curtis dissimilarities and Jaccard distances. Principal coordinate analysis (PCoA) based on the Jaccard and Bray-Curtis distance matrices were used to visualize the differences in community membership and structure, respectively. The nonparametric Wilcoxon Rank Sum test procedure in SAS (Cary, NC, United States) was used to determine significant differences between treatments within each age on Shannon diversity index and Observed OTUs (sows reared: SR; isolated reared: IR), while the analysis of similarities (ANOSIM) procedure was used to test the effect of age and treatments on the microbial community structure by using the ANOSIM command in mothur with the default settings (i.e., number of permutations = 1000). *P*-values were calculated based on the percent of times that the actual *R*-value surpassed the permutation-derived *R*′-values. Both classification (ranked by Mean Decrease Accuracy)- and regression (ranked by the percent increase in mean square error) based Random Forest analysis was performed to identify bacterial OTUs that are most predictive between the IR and SR groups and that are correlated with continuous variables such as BW by using R Random Forest 4.6-12 package (Liaw and Wiener, 2002).

Raw data were deposited into the SRA database with accession number PRINA488243.

### Flow Cytometry

Blood (3 ml) was collected into K2EDTA tubes from 1 pig per litter (10 litters per rearing strategy) on days 4, 11, 20, 32, 55, and 103. Peripheral blood mononuclear cells (PBMCs) were then isolated using Histopaque^®^1077 (Sigma-Aldrich, St. Louis, MO, United States) gradient centrifugation, and counted using a hemocytometer. Cell suspensions were incubated for 30 min in three combinations of staining antibodies with specificity for: (1) T lymphocytes [phycoerythrin (PE)-Cy7-conjugated mouse anti-pig CD3ε (clone BB23-8E6-8C8), peridinin-chlorophyll protein complex (PerCp)-Cy5.5-conjugated mouse anti-pig CD4 (Clone 74-12-4), and fluorescein isothiocyanate (FITC)-conjugated mouse anti-pig CD8α, all purchased from BD Biosciences, San Jose, CA), (2) monocytes/natural killer (NK) cells [mouse anti-pig CD16-PE (Clone G7), Novus Biologicals, Littleton, CO; mouse anti-pig CD14-FITC (Clone MIL2), Bio-Rad, Hercules, CA; with CD3ε-PE-Cy7], and (3) monocytes/dendritic cells [mouse anti-pig CD172a-PE (Clone 74-22-15A), BD Biosciences; with CD3ε-PE-Cy7, CD4-PerCP-Cy5.5, and CD14-FITC). Common components of these three stain sets were biotin-conjugated mouse anti-pig CD1 (Clone 76-7-4, Southern Biotech, Birmingham, AL, United States) as a marker for thymocytes, and LIVE/DEAD (LDA) fixable aqua dead cell stain (Affymetrix eBioscience, ThermoFisher Scientific, Waltham, MA, United States) to distinguish dead cells from viable cells.

To detect regulatory T (T_reg_) cells, cells were permeabilized and incubated with the Foxp3 staining solution (anti-mouse/rat Foxp3-PE, blocked with normal rat serum; Affymetrix eBioscience) following the manufacturer’s Instructions. Isotype control for Foxp3 was prepared with the monoclonal rat IgG2a, κ-PE (Affymetrix eBioscience) stain on pooled cell suspensions.

After completing the staining protocols, samples were fixed following manufacturer’s instructions (Affymetrix eBioscience) and stored in the dark at 4°C until immunophenotype data were acquired on a LSR II B-3 flow cytometer (BD Immunocytometry Systems, San Jose, CA, United States) at the University of Tennessee Flow Cytometry and Cell Sorting Laboratory, Memphis, TN, United States; data were analyzed with FlowJo Software (Tree Star, Inc., Ashland, OR, United States). The blood concentration (10^6^/ml) of various lymphocyte subsets was calculated based on the concentration of PBMCs in the cell suspension per ml of blood and the percentage of the various lymphocyte subsets in PBMC suspensions.

### Performance Measurements

As for performance, individual pig BW was measured at birth, weaning (day 21), at each subsequent phase change when pen feed intake was measured, and upon study completion (day 181) to determine average daily gain (ADG), average daily feed intake (ADFI), and feed efficiency (G:F) by pen.

Fat-O-Meat’er (Carometec A/S, Denmark) real-time ultrasound of 10th rib back fat (BF) depth and longissimus muscle (LM) area of individual pigs were measured at study completion to estimate carcass composition using the equation suggested by Burson (2006).

### Statistical Analyses

Data on growth performance and carcass traits were analyzed as randomized complete block design (RCBD) with ANOVA generated using the GLM procedure of SAS 9.3 (SAS Inst. Inc., Cary, NC, United States). The model included fixed effects of treatment, sex, and all appropriate interactions, and BW block was used as random effect. Data on immunophenotypes were analyzed as a randomized complete block design with ANOVA generated using the mixed procedure of SAS 9.3 (SAS Inst. Inc., Cary, NC, United States). In addition, immunophenotyping data were analyzed as repeated measures with age (day) as well as treatment by age interaction included in the model as fixed effects. Pen was the experimental unit for analysis of performance whereas individual pig was used for blood lymphocyte counts. Least squares means were calculated for all dependent variables and means separations were performed using F protected *t*–test (PDIFF option). Differences between means were considered significant when probabilities were less than 0.05 (*P* ≤ 0.05) or tendencies when 0.05 < *P* ≤ 0.10.

## Author Contributions

CM, MM, FC, and TT conceived the experiments. HK and TT collected the performance data and animal management. TT, MM, GE, MS, and XW performed the lab procedures. TT, MM, GE, and JZ analyzed the results. MS, TT, and JZ wrote the manuscript with input from all authors.

## Conflict of Interest Statement

The authors declare that the research was conducted in the absence of any commercial or financial relationships that could be construed as a potential conflict of interest.

## Footnotes

1 https://www.mothur.org/wiki/MiSeq_SOP
