# Supplementary material for: Isolated Rearing at Lactation Increases Gut Microbial Diversity and Post-weaning Performance in Pigs
Source: Front Microbiol. 2018 Nov 29;9:2889. doi: 10.3389/fmicb.2018.02889 (PMC6282802; doi:10.3389/fmicb.2018.02889)
Supplement: Supplementary file 1 [file Table_1.pdf]

Table S1 Body weight and average daily gain of pigs reared with the sow (SR) or in isolation (IR).

Item	Treatments			*P* - Value		
	CR (n = 40)	IR (n = 40)	SEM	Trt	Sex	Trt x Sex
**Body weight, kg**						
At birth (d 0)	1.24	1.27	0.042	0.565		
Weaned (d 21)	5.79	6.07	0.214	0.375		
Nursery						
End of Phase 1 (d 29)	5.61[x]	6.12[y]	0.193	0.078		
End of phase 2 (d 50)	11.88[a]	14.03[b]	0.408	**0.002**	0.140	0.568
End of phase 3 (d 62)	19.26[a]	22.15[b]	0.532	**0.002**	0.095	0.349
Grower						
End of phase 1 (d 85)	36.72[a]	39.46[b]	0.817	**0.032**	0.301	0.923
End of phase 2 (d 119)	69.45[a]	73.97[b]	1.506	**0.052**	0.911	0.492
Finisher						
End of phase 1 (d 141)	92.36[x]	96.94[y]	1.590	0.061	0.166	0.586
End of phase 2 (d 159)	112.39[x]	117.20[y]	1.900	0.095	0.122	0.601
End of phase 3 (d 181)	132.24[x]	136.37[y]	1.550	0.081	0.064	0.373
**Average daily gain, kg/d**						
Pre weaning	0.27	0.28	0.013	0.385		
NP1 (d 21-29)	-0.02	0.01	0.013	0.124		
NP2 (d 29-50)	0.30[a]	0.38[b]	0.015	**0.002**	0.175	0.572
NP3 (d 50-62)	0.61[a]	0.68[b]	0.014	**0.006**	0.074	0.112
G1 (d 62-85)	0.76	0.75	0.016	0.827	0.800	0.279
G2 (d 85-119)	0.96	1.02	0.028	0.216	0.294	0.326
F1 (d 119-140)	1.09	1.09	0.02	0.913	**< 0.001**	0.683
F2 (d 140-159)	1.05	1.07	0.031	0.786	0.200	0.835
F3 (d 159-181)	1.04	1.01	0.063	0.694	0.993	0.733
NP2 to NP3 (d 29-50)	0.41[a]	0.49[b]	0.014	**0.002**	0.114	0.328
Overall Grower (d 62-119)	0.88	0.91	0.021	0.341	0.350	0.639
Overall Finisher (d 119-181)	1.06	1.06	0.021	0.832	**0.032**	0.772
Overall Growing/finishing (d 62-181)	0.97	0.98	0.012	0.542	**0.012**	0.525
Overall (d 29-181)	0.85	0.87	0.010	0.110	**0.046**	0.376

Four littermates were selected 4 days postpartum from 20 sows with two neonates transferred to offsite nursery facility and housed in deck while 2 cohorts remained with sows during d 4 to 21. Upon weaning at d 21, pigs with the same lactation treatments and gender were housed in pens to have four pigs per pen, and ten pens per treatment. Data were analyzed using GLM procedure of SAS with lactation treatment and gender used as fix effect, and pen was experimental unit.

[a.b.] Row with different superscripts differ significantly ($P < 0.05$) [x.y.] Row with different superscripts tend to be differ ($P < 0.10$)
