# Supplementary material for: Isolated Rearing at Lactation Increases Gut Microbial Diversity and Post-weaning Performance in Pigs
Source: Front Microbiol. 2018 Nov 29;9:2889. doi: 10.3389/fmicb.2018.02889 (PMC6282802; doi:10.3389/fmicb.2018.02889)
Supplement: Supplementary file 2 [file Table_2.pdf]

Table S2. Average daily feed intake and feed efficiency of pigs reared with the sow (SR) or in isolation (IR).

	Treatments			*P* - Value		
	SR	IR	SEM	Trt	Sex	Trt x Sex
**Average daily feed intake, kg/d**						
NP1 (d 21-29)	0.10[a]	0.16[b]	0.009	**<0.0001**		
NP2 (d 29-50)	0.41[a]	0.51[b]	0.019	**0.003**	0.141	0.303
NP3 (d 50-62)	0.91[x]	1.01[y]	0.040	0.097	**0.050**	0.610
G1 (d 62-85)	1.68	1.63	0.113	0.726	0.191	0.416
G2 (d 85-119)	2.49	2.48	0.122	0.918	0.131	0.638
F1 (d 119-140)	3.14	3.09	0.137	0.792	**0.022**	0.733
F2 (d 140-159)	3.35	3.27	0.125	0.678	0.064	0.962
F3 (d 159-181)	3.46	3.15	0.133	0.119	0.105	0.480
NP2 to NP3 (d 29-50)	0.59[a]	0.61[b]	0.026	**0.017**	0.070	0.435
Overall Grower (d 62-119)	2.17	2.13	0.103	0.820	0.614	0.491
Overall Finisher (d 119-181)	3.31	3.17	0.121	0.420	**0.034**	0.896
Overall Growing/finishing (d 62-181)	2.74	2.66	0.102	0.546	0.117	0.672
Overall (d 29-181)	2.27	2.22	0.083	0.689	0.164	0.725
**Gain-to-feed ratio**						
NP1 (d 21-29)	-0.44	0.04	0.199	0.110		
NP2 (d 29-50)	0.71	0.70	0.017	0.486	0.851	0.403
NP3 (d 50-62)	0.68[b]	0.64[a]	0.015	**0.053**	0.116	0.343
G1 (d 62-85)	0.47	0.45	0.029	0.691	0.274	0.549
G2 (d 85-119)	0.39	0.39	0.014	0.900	0.261	0.076
F1 (d 119-140)	0.34	0.32	0.010	0.249	0.508	0.669
F2 (d 140-159)	0.34	0.33	0.009	0.553	0.176	0.643
F3 (d 159-181)	0.27	0.26	0.019	0.883	0.348	0.486
NP2 to NP3 (d 29-50)	0.70[b]	0.66[a]	0.011	0.055	0.214	0.889
Overall Grower (d 62-119)	0.41	0.41	0.016	0.750	0.926	0.191
Overall Finisher (d 119-181)	0.31	0.30	0.010	0.595	0.246	0.592
Overall Growing/finishing (d 62-181)	0.35	0.34	0.010	0.657	0.354	0.264
Overall (d 29-181)	0.37	0.36	0.010	0.729	0.272	0.273
**Carcass traits**						
10th rib back fat depth, mm	20.00	23.09	1.00	**0.046**	**0.000**	0.680
10th rib longissimus muscle area, cm^2	47.21	49.50	0.85	0.079	0.127	0.984
Lean, %	38.52	38.21	0.35	0.539	**< 0.0001**	0.833

[a.b.] Row with different superscripts differ significantly ($P < 0.05$)
[x.y.] Row with different superscripts tend to be differ ($P < 0.10$)
