# Supplementary material for: Isolated Rearing at Lactation Increases Gut Microbial Diversity and Post-weaning Performance in Pigs
Source: Front Microbiol. 2018 Nov 29;9:2889. doi: 10.3389/fmicb.2018.02889 (PMC6282802; doi:10.3389/fmicb.2018.02889)
Supplement: Supplementary file 3 [file Table_3.pdf]

Table S3. Bacteria operational taxonomy units (OTUs) that are most correlated with dependent variables.

Variables	OTUs		Correlation coefficient	*P* - Value
day 50 BW	224	Fusicatenibacter	0.29	0.02
	389	Lactococcus	0.29	0.02
day 62 BW	224	Fusicatenibacter	0.32	0.01
ADG 2129	53	Blautia	0.33	0.01
ADG 2950	11	Leuconostoc	0.33	0.01
	389	Lactococcus	0.33	0.01
ADG 2162	11	Leuconostoc	0.33	0.01
day 21 Foxp3^{+} T_{reg}	389	Lactococcus	-0.27	0.02
	11	Leuconostoc	-0.24	0.04

Individual pigs' fecal samples were collected on day 20, for sequencing of the V4 hypervariable region of 16S rRNA. Sequences were analyzed using mothur (1.34.0) pipeline. Regression based (ranked by the percent increase in mean square error) random forest analysis was used (Random Forest 4.6-12 package). Pearson correlation analysis was used to test correlation between OTUs and continuous variables (R). BW: body weight; ADG: average daily gain.
