# Supplementary material for: Isolated Rearing at Lactation Increases Gut Microbial Diversity and Post-weaning Performance in Pigs
Source: Front Microbiol. 2018 Nov 29;9:2889. doi: 10.3389/fmicb.2018.02889 (PMC6282802; doi:10.3389/fmicb.2018.02889)
Supplement: Supplementary file 4 [file Table_4.pdf]

Table S4. Age by treatment interaction effect on innate and adaptive peripheral blood leukocytes count (x10^6/ml blood) in sows reared (SR) or isolated reared (IR).

	Sow reared (SR)						Isolated reared							P - Value
	d 4	d 11	d 20	d 32	d 55	d 103	d 4	d 11	d 20	d 32	d 55	d 103	SEM	Age*Trt
PBMC cell count	24.97	87.45	62.36	5.16	7.27	8.56	27.13	27.72	21.50	5.62	6.15	7.66	7.04	<0.01
$CD3^+$ T cells	7.55	37.61	29.17	2.34	3.95	5.46	6.67	10.02	11.34	2.56	3.25	4.56	3.6	< 0.01
$CD3^+CD8^+$ T cells	0.82	3.71	4.68	0.26	0.95	0.87	0.65	0.75	1.48	0.28	0.75	0.63	0.45	< 0.01
$CD3^+CD4^+$ T cells	4.16	16.58	16.15	1.29	1.50	1.55	3.57	4.79	7.19	1.50	1.27	1.42	1.72	< 0.03
$CD4^+$:$CD8^+$ ratio	5.89	5.40	3.82	5.35	2.22	2.01	5.85	7.66	5.36	5.35	2.69	2.35	0.39	0.01
$CD3^+CD4^+Foxp3^+$ Treg	0.38	1.05	0.72	0.05	0.04	0.07	0.35	0.15	0.20	0.06	0.03	0.05	0.11	< 0.01
$CD3^{neg}CD4^{neg}CD172^+$	4.61	7.01	2.90	0.31	0.68	0.51	3.44	3.61	1.81	0.30	0.59	0.42	0.78	0.23
$CD14^+$	3.44	4.27	2.21	0.23	0.49	0.19	2.67	2.19	1.42	0.17	0.36	0.16	0.51	0.32

One pig from each litter (10 litter per treatments) were selected for blood sampling. An attempt was made to sample the same pig throughout the entire study. The whole blood samples (3 mL) were obtained at d 4 (initial), 11, 20 (weaning), 32, 55, and 103 of ages. PBMCs were isolated using Histopaque®1077 (Sigma-Aldrich, St. Louis, MO) density gradient centrifugation, and total PBMCs isolated determined using hemacytometer. Cell suspensions in 100 µl of PBS + 2% FBS buffer were incubated in staining antibodies with surface markers for T cells (mouse anti-pig CD3ε-PECy7, mouse anti-pig CD4-PerCp-Cy5.5, and mouse anti-pig CD8α-FITC; BD Biosciences, San Jose, CA), the intracellular protein Foxp3 (anti-mouse/rat Foxp3-PE; ThermoFisher Scientific, Waltham, MA) as a marker for T_{reg} cells, and for monocytes/dendritic cells/NK cells (mouse anti-pig CD172a-PE, BD Biosciences; mouse anti-pig CD14-FITC, Bio-Rad, Hercules, CA). In addition, a live/dead aqua stain was also included with all staining combinations to determine cell viability.
