# Supplementary material for: Isolated Rearing at Lactation Increases Gut Microbial Diversity and Post-weaning Performance in Pigs
Source: Front Microbiol. 2018 Nov 29;9:2889. doi: 10.3389/fmicb.2018.02889 (PMC6282802; doi:10.3389/fmicb.2018.02889)
Supplement: Supplementary file 5 [file Table_5.pdf]

Table S5. Nursery, grower and finisher diets composition (%, as-fed basis)

Phase	Nursery			Grower		Finisher		
	1	2	3	1	2	1	2	3
Duration, d/d	21/29	30/50	51/62	62/85	85/119	119/141	141/159	159/181
Ingredients, %								
Corn	34.93	42.69	47.04	54.38	52.75	57.27	60.24	78.11
Soybean meal	11.25	22.5	28	21.9	13.75	9.4	6.5	8
Corn DDGS[1]	7.5	10	16.25	20	30	30	30	10
Fat	2.5	2.5	2.5	1	1	1	1	1
MonoCa-P[2]			0.075	0.14				0.15
Limestone	0.2	0.525	0.77	1.26	1.3	1.25	1.175	1.1
Salt	0.2	0.35	0.5	0.6	0.5	0.5	0.5	0.975
L-Lysine	0.265	0.3	0.3	0.33	0.33	0.3	0.3	0.3
DL-Methionine	0.153	0.139	0.085					
L-Threonine	0.049	0.056	0.036	0.036	0.007			0.076
L-Tryptophan	0.01			0.003	0.010	0.010	0.013	0.017
Trace Mineral Premix[3]	0.15	0.15	0.15	0.15	0.15	0.1	0.1	0.1
Vitamin Premix[4]	0.25	0.25	0.25	0.15	0.15	0.125	0.125	0.125
SDPP[5]	3.5	1.5						
Milk Whey	25	12						
Other[6]	14.045	7.045	4.045	0.0485	0.0485	0.0485	0.0485	0.0485
Total	100	100	100	100	100	100	100	100
Calculate:								
ME[7], Mcal/kg	3.480	3.434	3.401	3.35	3.36	3.37	3.37	3.35
CP[8], %	22.97	24.45	24.68	20.8	19.5	17.8	16.6	13.4
SID Lysine, %	1.46	1.42	1.28	1.05	0.88	0.75	0.68	0.65
Available P, %	0.50	0.35	0.23	0.19	0.18	0.17	0.17	0.14
Ca, %	0.85	0.75	0.65	0.61	0.58	0.54	0.51	0.50
SID M+C:Lys	58.1	58.0	58.0	55.3	62.9	68.7	72.2	63.2
SID Thr:Lys	60.1	60.0	60.1	61.1	62.0	64.0	64.9	68.1
SID Trp:Lys	17.1	17.2	17.9	18.0	18.1	18.1	18.0	18.0

1. Corn distillers dried grain contains soluble with 5 to 9 % of fat. 2. Mono calcium phosphate. 3. Supplied 11023 IU vitamin A, 1653 IU vitamin D_3 as D-activated animal sterol, 44 IU vitamin E, 4.4 mg vitamin K as menadione sodium bisulfite complex, 33 mg pantothenic acid as D-calcium pantothenate, 55 mg niacin, 10 mg riboflavin, and 44 μg vitamin B_{12} per kilogram of feed. 4. Supplied 0.30 mg Se as sodium selenite, 40 mg Mn as manganous oxide, 165 mg Zn as zinc oxide, 165 mg Fe as ferrous sulfate, 17 mg Cu as copper sulfate, and 0.30 mg of I as calcium iodate per kilogram of feed. 5. Spray dried porcine plasma (AP920®; APC, Inc., Ankeny, IW). 6 A 0.015% and 0.019 % phytase (Ronozyme® P CT; DSM Nutritional Products, Inc., Parsippany, NJ) were added in nursery and grower/finisher phases respectively; Feed preservative (Quinguard®; Novus International, Inc., St. Louis, MO) were supplemented at 0.03% in each phase; Milk lactose was provided at 3.5 % in nursery phase 1 only. Poultry by product was added at 4% throughout nursery phases. Fish meal was added at 6.5 and 3 % in nursery phase 1 and 2, respectively.
